# Atypical Presentation of Non-small Cell Lung Carcinoma

**DOI:** 10.7759/cureus.81899

**Published:** 2025-04-08

**Authors:** Ammaar Jan, Oudhay S Sohal, Delaram Pourkalbassi, Desiree Boisson, Nick Taylor, Lorena Rivera, Ernest Metelus, Damian Casadesus

**Affiliations:** 1 Internal Medicine, Jackson Memorial Hospital, Miami, USA; 2 Internal Medicine, American University of the Caribbean School of Medicine, Cupecoy, SXM; 3 Internal Medicine, Ross University School of Medicine, Bridgetown, BRB; 4 Internal Medicine, St. George’s University School of Medicine, St. George’s, GRD; 5 Internal Medicine, St. George’s University School of Medicine, St. George's, GRD

**Keywords:** cardiac tamponade, lung, metastasis, non-small cell lung carcinoma (nsclc), pituitary gland

## Abstract

The typical presentation of non-small cell lung carcinoma (NSCLC) is hemoptysis, dyspnea, and chest pain. A late cancer diagnosis commonly leads to the patient presenting with metastasis to the bone, liver, adrenal glands, or brain. We present a 58-year-old man who visited the emergency department with a headache for two weeks. A CT scan of the brain revealed a right posterior parietal mass with surrounding vasogenic edema and sulcal effacement as well as a large mass arising from the sella. The patient underwent mass resection, and the pathology results indicated a metastatic adenosquamous carcinoma of the lung. The patient later returned to the emergency department with dyspnea and a cough, and a thorough work-up indicated the patient was suffering from cardiac tamponade secondary to metastasis. The patient underwent a pericardiectomy, pericardial window, and thoracentesis with thoracostomy tube placement. One liter of pericardial fluid was removed, and he was discharged with follow-up for outpatient treatment. Overall, this patient has a poor prognosis due to the presentation of metastases to the brain and pericardium.

This case underscores the importance of considering NSCLC in patients with unusual central nervous system presentations and highlights the rare but critical manifestations of pituitary and pericardial metastases. Given the poor prognosis associated with metastatic cardiac tamponade and pituitary involvement, early recognition and symptom management are essential to optimizing patient outcomes.

## Introduction

Lung cancer is the primary cause of cancer-related deaths globally, with the majority being a form of non-small cell lung carcinoma (NSCLC) [1,2]. The most common initial presenting symptoms of NSCLC are hemoptysis, dyspnea, and chest pain [3]. While various malignancies of the lung, breast, prostate, kidneys, and colon have been reported to metastasize to the pituitary, lung cancer is the most common source [4]. However, pituitary metastasis is still overall quite rare, occurring in less than 1% of metastatic lesions [5]. Most pituitary metastases are asymptomatic, with characteristic symptoms reported in less than 20% of cases. Moreover, most of these tumors are often incidentally identified during autopsy or in patients with advanced malignancy. Pituitary metastasis has also seldom been the initial manifestation of an unknown primary tumor, occurring in only 20-30% of cases [5].

In addition, although cardiac metastasis from cancer is not uncommon, malignant pericardial effusion is frequently diagnosed post-mortem, and cardiac tamponade remains a rare presentation of any malignancy [6,7]. Cancers that commonly cause pericardial effusion include lung cancer, breast cancer, and gastrointestinal malignancies [8].

We present a unique case of a 58-year-old man who initially presented to the hospital with a seizure and was found to have a pituitary metastasis and later cardiac tamponade from a non-small cell lung carcinoma.

## Case presentation

A 58-year-old man with no significant medical history initially presented to the hospital with complaints of a headache for two weeks, followed by a seizure with a postictal state. The patient had no family history of cancer. He stopped smoking more than 25 years prior to his hospital admission, and denied the use of alcohol and recreational drugs. On arrival his vital signs were a temperature (T) of 36.9 C, heart rate (HR) of 89 beats per minute (bpm), respiratory rate (RR) of 20 breaths per minute (brpm), blood pressure (BP) of 128/82 mmHg, and oxygen saturation 100% on ambient air. After recovery from his seizure, the patient was awake, alert, and oriented to person, time, place, and situation. The cardiopulmonary and abdominal examinations were normal. Neurological examination revealed bitemporal hemianopsia.

**Figure 1:**
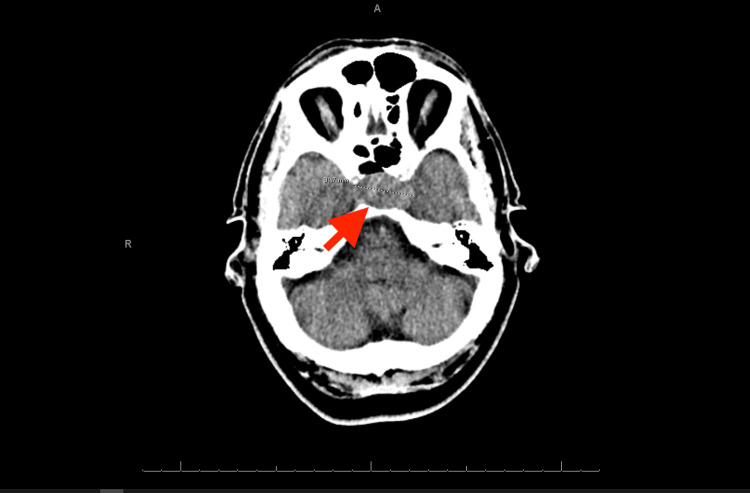
CT scan of the brain with a large mass arising from the sella measuring 3.2 x 2.2 cm.

**Figure 2:**
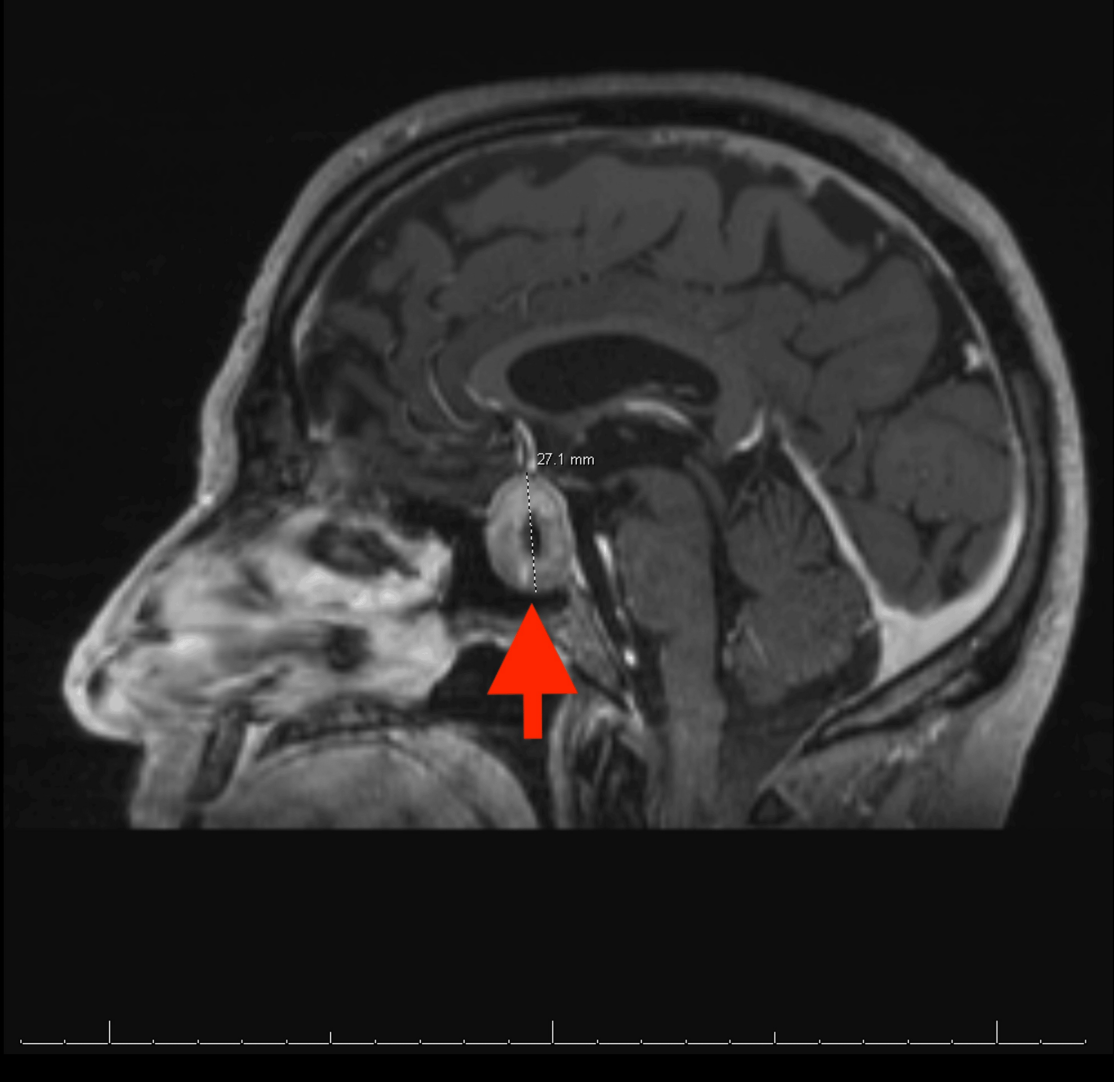
MRI of the brain showing  an expansile sellar and suprasellar mass measuring 1.9 x 2.4 x 2.7 cm.

A computed tomography (CT) scan of the brain revealed a right posterior parietal mass measuring 1.8 cm in size with surrounding vasogenic edema and sulcal effacement as well as a large mass arising from the sella measuring 3.2 x 2.2 cm in size (Figure 1). Magnetic resonance imaging (MRI) of the brain revealed a heterogeneously enhancing expansile sellar and suprasellar mass with central hypoattenuation measuring 1.9 x 2.4 x 2.7 cm (Figure 2). The CT scan of the chest revealed small right-sided pulmonary nodules. Considering the patient’s age, the small size of the nodules, and that he quit smoking more than 25 years ago, it was initially thought that the pituitary mass may have been the primary tumor.

**Figure 3:**
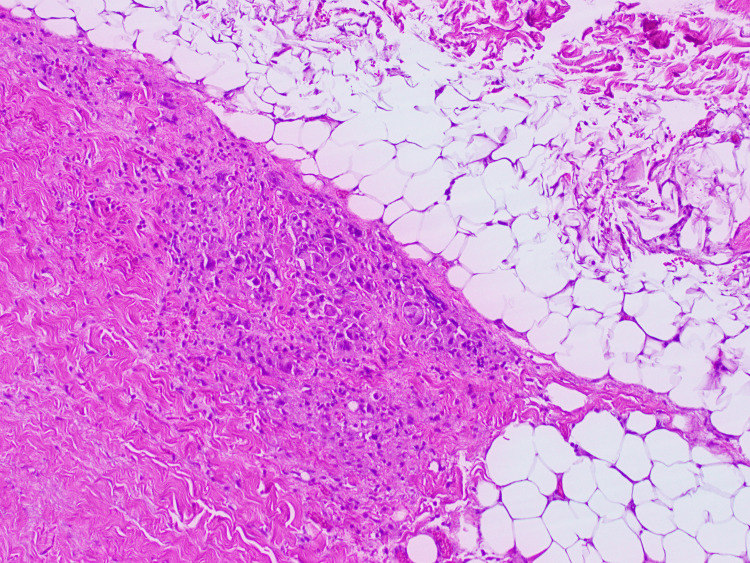
Metastatic lung adenosquamous carcinoma.

**Figure 4:**
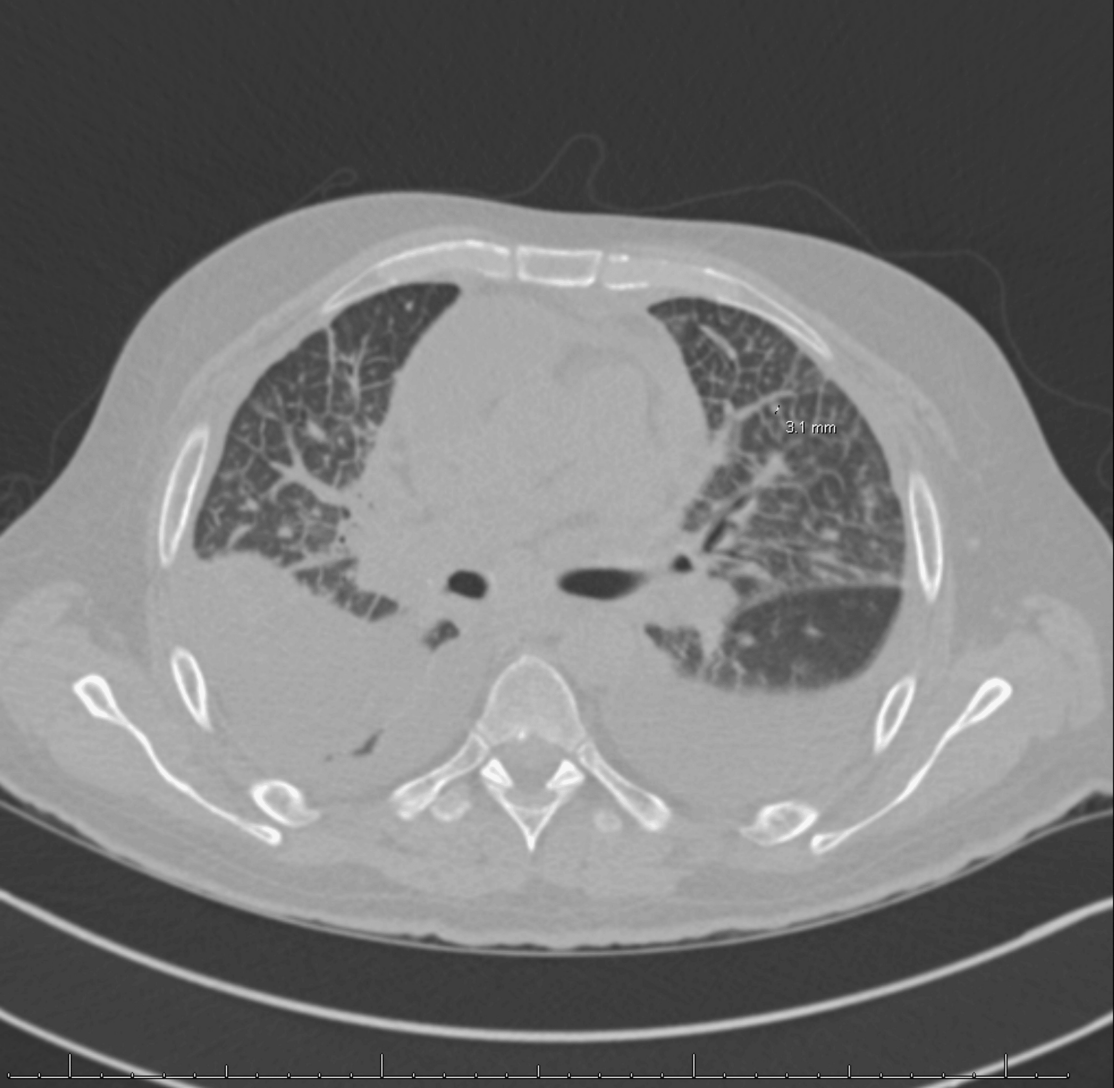
Repeat CT scan of the chest revealing diffuse severe pulmonary edema, large volume left and multioculated moderate volume right pleural effusion with left lower lobe and lingular atelectasis, mediastinal and right supraclavicular lymphadenopathy, multiple pulmonary nodules measuring up to 6 mm, and moderate pericardial effusion.

The patient was scheduled for posterior parietal mass resection, but his surgery had to be delayed due to perforated diverticulitis, requiring exploratory laparotomy and sigmoidectomy. Once he was stable, he underwent an occipital craniotomy for resection of the posterior parietal mass. The biopsy was positive for cytokeratin, TTF-1, Napsin A, p63, and negative for CK20, GATA-3, and PAX-8, indicating a metastatic lung adenosquamous carcinoma as the primary lesion (Figure 3). A repeat CT scan of the chest revealed bilateral ground-glass opacities and interlobular septal thickening, multiple bilateral pulmonary nodules, with the largest one measuring 7 mm, and stable mediastinal and hilar lymphadenopathy (Figure 4). 

The patient was discharged with plans for outpatient pituitary tumor resection, but he presented to the hospital once again with dyspnea and cough. Vital signs in the emergency department were T of 36.6 C, HR of 114 bpm, RR of 20 brpm, BP of 96/82 mmHg, and oxygen saturation of 100% on ambient air. 

The laboratory values obtained are shown in Table 1.

**Table 1:** Laboratory values FSH: follicle-stimulating hormone; HGH: human growth hormone; LH: luteinizing hormone; IGF: insulin-like growth factor; TSH: thyroid-stimulating hormone; ACTH: adrenocorticotropic hormone

Laboratory test	Patient laboratory value	Reference range
Glucose	136 mg/dL (high)	74-106 mg/dL
Sodium	136 mmol/L (low)	137-145 mmol/L
Chloride	109 mmol/L (high)	98-107 mmol/L
Anion gap	5 mEq/L (low)	7-15 mEq/L
Albumin	3.8 g/dL (low)	3.9-5.0 g/dL
WBC count	12.1x10^3/mcL (high)	4.0-10.5 x 10^3/mcL
Hemoglobin	13.1 g/dL (low)	Men: 13.3-16.3 g/dL
Hematocrit	38.3% (low)	Men: 39-47.1%
Neutrophil %	76.3% (high)	36-70%
Lymphocyte %	14.3% (low)	16-43%
Absolute neutrophils	9.3x10^3/mcL (high)	2.0-6 x 10^3/mcL
Cortisol	14.9 mcg/dL	4.3-22.4 mcg/dL
FSH	4 mIU/mL	1.0-8.0 mIU/mL
Free T4	1.35 ng/dL	0.93-1.70 ng/dL
HGH	0.1 ng/mL	< OR=7.1 ng/mL
LH	4.3 mIU/mL	1.7-7.7 mIU/mL
Prolactin	15.2 ng/mL	4.0-15.2 ng/mL
IGF-1	99 ng/mL	50-317 ng/mL
TSH	2.190 mcIU/mL	0.270-4.200 mcIU/mL
ACTH	28.6 pg/mL	7.2-63.3 pg/mL

**Figure 5:**
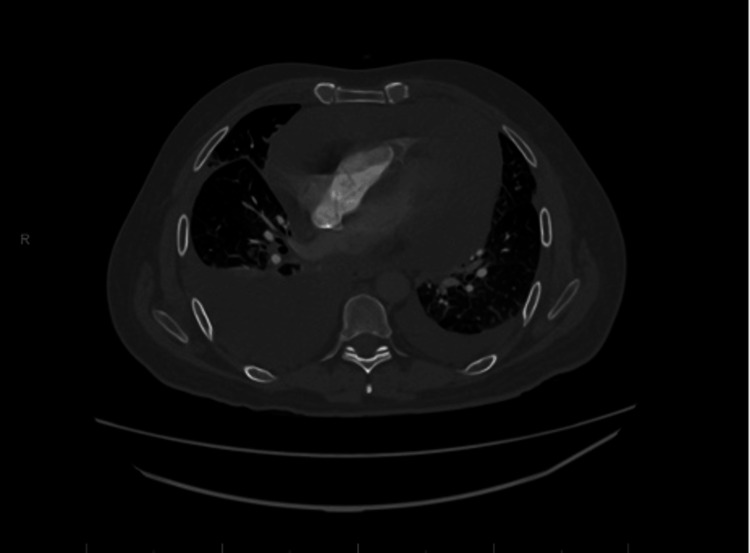
CT scan of the chest demonstrating multiple pulmonary nodules, diffuse ground-glass opacities, interlobular septal thickening, bilateral effusions and a large pericardial effusion with a mass effect on the cardiac chambers.

On physical examination, bilateral crackles were auscultated. A chest x-ray revealed an enlarged cardiac silhouette with bilateral pleural effusions. The CT scan of the chest demonstrated multiple pulmonary nodules, diffuse ground-glass opacities, interlobular septal thickening, bilateral effusions, and a large pericardial effusion with a mass effect on the cardiac chambers (Figure 5).

The patient underwent a pericardiectomy, pericardial window, and thoracentesis with thoracostomy tube placement. One liter of pericardial fluid was removed, and the pathology results were positive for TTF-1, indicating metastatic disease. The patient was discharged and was scheduled to follow up as an outpatient with neurosurgery for pituitary mass resection and with oncology and radiation oncology for NSCLC treatment. The patient did not return to the emergency department or the outpatient clinic.

## Discussion

Early indicators of NSCLC can present as pulmonary nodules that can determine a malignant course based on the size of the nodule, age, smoking history, family history of lung cancer, exposure to environmental/occupational hazards, location, and calcification. Nodules larger than 8 mm are more likely to be malignant and are often carefully monitored over the course of a disease. Individuals over the age of 50 are also considered at risk of malignant nodules if present on imaging. Current or former smokers, as well as those exposed to environmental/occupational hazards, have a significantly higher likelihood of malignant nodules. Nodules in the upper lobes of lungs, as well as calcification patterns (such as non-calcified or irregularly calcified nodules), in comparison to a benign pattern of calcification (such as central, popcorn, or concentric) should be closely assessed [8]. However, when evaluating NSCLC diagnoses, symptoms such as headaches, cognitive changes, or focal neurological deficits are often initial symptoms. Amongst the symptoms of NSCLC, seizures are often uncommon. Seizures occur in approximately 10-30% of patients with brain metastases, but are often following the development of other symptoms [9]. 

Cases where NSCLC has metastasized to the pericardium and the pituitary require an understanding of its impact on the prognosis, how to guide treatment decisions, symptom management, monitoring disease progression, decision-making for palliative care, and understanding the underlying cancer biology. 

Metastatic cardiac tamponade is considered a poor prognostic indicator because of its advanced stage of cancer, aggressive tumor type, complications associated with fluid accumulation in the pericardium, limited treatment options, systemic involvement, and overall survival rate. Once signs of cardiac tamponade are present, it is an indication that widespread metastatic disease already exists, the tumor burden is high, and the disease course is rapidly progressing. Cardiac tamponade is often an indication of the terminal phase of a disease and can complicate cancer treatment due to the urgent need to manage the tamponade [10]. In cases of NSCLC, the cancer is usually at a stage where it is no longer responsive to treatment; hence, the patient is more likely to succumb to complications such as cardiac tamponade or other systemic involvement [11]. 

Studies have shown that the median survival time after diagnosis of cardiac metastasis can be as short as 7.48 months, with a significant drop in survival if the patient does not receive continued treatment. The one-year survival rate is less than 40%, and the median survival without treatment drops to about 2.1 months [12]. While pericardiocentesis can temporarily relieve symptoms, it does not address the underlying cancer. In addition, the recurrence of tamponade is common, and treatment options become increasingly limited as the disease progresses. Individuals may undergo the pericardial window technique in situations with recurrent and malignant pericardial effusion. Although more invasive, this technique makes an incision below the sternum, giving the pericardium a window to drain fluid into the abdominal cavity. This provides individuals with a long-term solution with low recurrence rates as fluid can drain freely and not build up in the pericardium [13]. 

Pituitary metastasis is also considered to be a poor prognostic indicator because of its advanced stage of disease, associated cancers, complex clinical management, and prognostic statistics. The condition is often seen in cancers that have spread extensively, particularly from primary tumors in the breast, lung, or kidney. Metastasis to sites other than the pituitary is a point of concern due to potential invasion of critical structures within the central nervous system, leading to symptoms such as headaches, visual disturbances, and cranial nerve deficits. Due to its advanced, disseminated malignancy, the overall prognosis is generally poor, with median survival after diagnosis ranging from a few months to over a year [14]. Studies have shown that the median survival time after detecting pituitary metastasis is about 11 months, with only 49% at one year and 37% of patients surviving at two years post-diagnosis. In addition to the advanced stages of the disease, symptom burden also plays a significant role [15]. Although our patient did not present with any endocrine dysfunction, some individuals can begin to experience a decline in functional status and higher reliance on palliative care because of an influx in endocrine dysfunctions such as adrenal insufficiency, hypothyroidism, diabetes insipidus, and hypogonadism. Treatment for pituitary metastasis is case by case and includes, but is not limited to transsphenoidal surgery, stereotactic radiosurgery, external beam radiation therapy, chemotherapy, and immunotherapy.

## Conclusions

Our case highlights an atypical presentation of NSCLC as well as the complex challenges posed by pituitary metastasis and metastatic cardiac tamponade in advanced stages. Both conditions indicate poor prognosis due to the aggressive nature of the underlying malignancy, significant tumor burden, and limited treatment options. The patient’s presentation of pituitary and pericardial involvement warranted involvement of oncology for treatment planning and the initiation of systemic therapy. Early identification and management of symptoms related to these metastases, such as pericardiocentesis, pericardial window, and surgical resection, can help alleviate the immediate clinical impact but do not substantially alter the disease course. Given the rapid progression and widespread systemic involvement, palliative care remains a key component of management, focusing on improving quality of life. 
